# Predicting atrial fibrillation episodes with rapid ventricular rates associated with low levels of activity

**DOI:** 10.1186/s12911-021-01723-3

**Published:** 2021-12-28

**Authors:** Zhi Li, Kevin M. Wheelock, Sangeeta Lathkar-Pradhan, Hakan Oral, Daniel J. Clauw, Pujitha Gunaratne, Jonathan Gryak, Kayvan Najarian, Brahmajee K. Nallamothu, Hamid Ghanbari

**Affiliations:** 1 Department of Computational Medicine and Bioinformatics, University of Michigan, Ann Arbor, MI USA; 2 Department of Internal Medicine, Yale School of Medicine, New Haven, CT USA; 3 Department of Internal Medicine, Division of Cardiovascular Medicine, Cardiac Electrophysiology Services, 1500 East Medical Center Drive, 48109-5853 Ann Arbor, Michigan USA; 4 Department of Anesthesiology, University of Michigan Medical School, Ann Arbor, MI USA; 5 Michigan Institute for Data Science, University of Michigan, Ann Arbor, MI USA; 6 Department of Emergency Medicine, University of Michigan, Ann Arbor, MI USA; 7 Michigan Center for Integrative Research in Critical Care, University of Michigan, Ann Arbor, MI USA; 8 Toyota Motor North America, Ann Arbor, MI USA

**Keywords:** Machine learning, Atrial fibrillation, Arrhythmia prediction, Signal processing, Probabilistic finite-state automata

## Abstract

**Background:**

Rapid and irregular ventricular rates (RVR) are an important consequence of atrial fibrillation (AF). Raw accelerometry data in combination with electrocardiogram (ECG) data have the potential to distinguish inappropriate from appropriate tachycardia in AF. This can allow for the development of a just-in-time intervention for clinical treatments of AF events. The objective of this study is to develop a machine learning algorithm that can distinguish episodes of AF with RVR that are associated with low levels of activity.

**Methods:**

This study involves 45 patients with persistent or paroxysmal AF. The ECG and accelerometer data were recorded continuously for up to 3 weeks. The prediction of AF episodes with RVR and low activity was achieved using a deterministic probabilistic finite-state automata (DPFA)-based approach. Rapid and irregular ventricular rate (RVR) is defined as having heart rates (HR) greater than 110 beats per minute (BPM) and high activity is defined as greater than 0.75 quantile of the activity level. The AF events were annotated using the FDA-cleared BeatLogic algorithm. Various time intervals prior to the events were used to determine the longest prediction intervals for predicting AF with RVR episodes associated with low levels of activity.

**Results:**

Among the 961 annotated AF events, 292 met the criterion for RVR episode. There were 176 and 116 episodes with low and high activity levels respectively. Out of the 961 AF episodes, 770 (80.1%) were used in the training data set and the remaining 191 intervals were held out for testing. The model was able to predict AF with RVR and low activity up to 4.5 min before the events. The mean prediction performance gradually decreased as the time to events increased. The overall Area under the ROC Curve (AUC) for the model lies within the range of 0.67–0.78.

**Conclusion:**

The DPFA algorithm can predict AF with RVR associated with low levels of activity up to 4.5 min before the onset of the event. This would enable the development of just-in-time interventions that could reduce the morbidity and mortality associated with AF and other similar arrhythmias.

## Introduction

$\hbox {Rapid and irregular ventricular rates (RVR)}$ are an important consequence of $\hbox {atrial fibrillation (AF)}$ [1]. A fast and irregular heart rate decreases time spent in diastole which impairs myocardial perfusion, ventricular diastolic filling, and cardiac output that in turn leads to greater symptom burden and reduced left ventricular systolic function [2, 3]. However, there are many reasons for RVR during episodes of AF. In addition to inappropriate tachycardia due to the underlying disease, patients’ heart rates may rise in a normal compensatory response to keep up with physiological demands during exercise or activities of daily living.

There is a need to not only detect and predict periods of AF with rapid ventricular rates but also distinguish periods of inappropriate tachycardia with AF from those associated with activity. Accelerometers are widely used in wearable sensors and provide important data that can be used to estimate patients’ activity and movement within their natural setting [4]. They can provide important insights that are not available through investigating $\hbox {electrocardiograms (ECGs)}$ alone. Therefore using raw accelerometry data in combination with ECG has the potential to identify and predict inappropriate tachycardia with AF. Accurate prediction of these episodes will allow just-in-time interventions for patients with AF.

Traditional algorithms have focused on the detection of AF episodes and are able to achieve this with high degree of accuracy [5]. However, prediction algorithms have not been able to achieve sufficient lag time to allow for medical or surgical interventions. There are several clinical scenarios where identifying rapid AF at low levels of activity can be of clinical value, including ensuring appropriate rate control for patients with AF, triggers for on-demand rhythm control approaches to improve patient care and facilitate monitoring system for AF that could be treated medically.

The aim of this study is to predict the onset of AF with RVR episodes associated with low activity using pre-event ECG signals. The proposed approach involves a DPFA-based algorithm that can predict episodes of AF with RVR that are associated with low levels of activity.

**Fig. 1:**
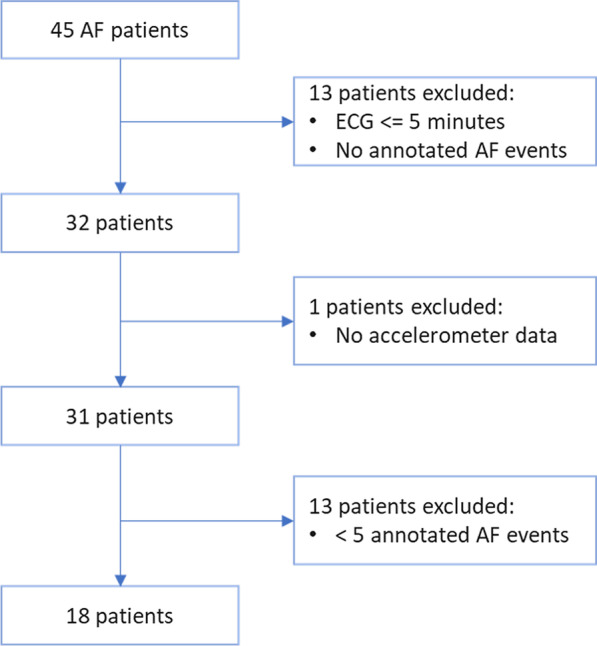
Patient flow

**Fig. 2:**
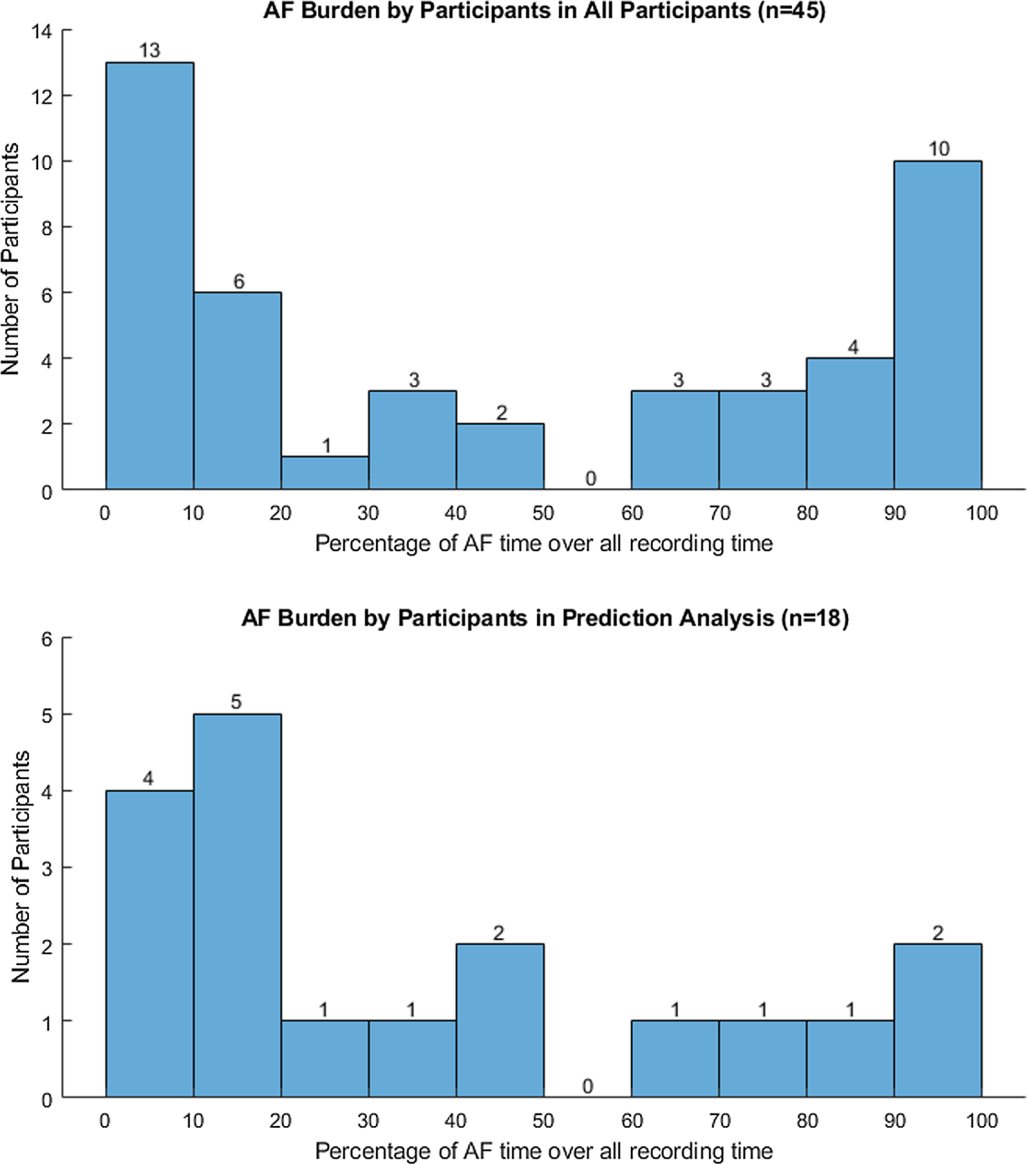
AF burden by participants. AF burden for all participant and those included in the prediction algorithm analysis

**Table.1:** Characteristics of patients

Variable	All participants (n=44*)	Participants in prediction analysis (n=18)
Female	14 (31.8%)	5 (27.8%)
Age	66.4 (11.7)	69.1 (7.3)
BMI	31.3 (6.1)	30.9 (5.7)
Hypertension	26 (591%)	11 (61.1%)
History of stroke	0 (0%)	0 (0%)
Diabetes	12 (27.3%)	5 (27.8%)
Coronary artery disease	11 (25%)	5 (27.8%)
Peripheral vascular disease	2 (4.5%)	2 (11.1%)
Beta blockers	31 (70.5%)	13 (72.2%)
Calcium channel blockers	14 (31.8%)	4 (22.2%)
Antiarrhythmic drugs	9 (20.5%)	2 (11.1%)

**Table.2:** AF episodes by activity level and HR level

	Low activity	High activity	Total
RVR	176	116	292
Non-RVR	545	124	669
Total	721	240	961

## Methods

A total of 45 patients with history of AF who presented to University of Michigan are recruited in the study. All patients wore an event recorder (Preventice solutions Inc) for up to 3 weeks. IRB approved the protocol and written informed consent was obtained. The ECG and accelerometer data were recorded continuously for up to 3 weeks. Thirteen patients are excluded because the length of the ECG is less than 5 min or there are no annotated AF events, 1 additional patient is excluded due to missing accelerometer data. Thirteen patients with AF episodes of duration less 30 s or less than 5 episodes of AF are  further excluded. Finally a total of 18 patients are included in the prediction analysis. A flow chart of the patient inclusion/exclusion criteria is demonstrated in Fig. 1. The characteristics of patients are summarized in Table 1. The demographic data is missing for 1 patient.

### Data sources

#### Accelerometer data

The raw accelerometer data are collected along three orthogonal axes in the device-specific frame of reference. The continuous accelerometer data are collected using a wireless monitoring device that adheres to patient’s chest (Preventice Solutions, Inc) and sampled at 10 Hz. The accelerometer data were up-sampled to 256 Hz to match the ECG sampling rate. For the analysis, the accelerometer magnitude *am*(*t*) consisting of the vector magnitude of the accelerometer data at each time point is used in analysis together with the synchronized ECG signal.

1 $$\begin{aligned} \begin{aligned} am(t)&=\sqrt{x(t)^{2}+y(t)^{2}+z(t)^{2}}\\&=\text {accelerometer magnitude at the time point } t. \end{aligned} \end{aligned}$$

The data is aggregated using $\hbox {mean amplitude deviation (MAD)}$ which computes the deviation of *am*(*t*) from its mean over the corresponding epoch, averaged over the length of the annotated AF signal [6].

2 $$\begin{aligned} MAD=\frac{1}{n}\times \sum _{i=1}^{n}\left| am(t_i)-{\overline{am}} \right| \end{aligned}$$

where:

$$\begin{aligned} \begin{aligned} am(t_{i})&=\text {accelerometer magnitude at the } i\text {th time point}\\ {\overline{am}}&=\text {mean accelerometer magnitude within the time period of interest}\\ n&=\text {length of the time period} \end{aligned} \end{aligned}$$

The $MAD \le 0.75$ quantile relative to the measurements from the entire group is designated as low activity and $MAD > 0.75$ quantile as high activity in order to account for inter-individual differences in activity levels within the study group. Threshold of 0.75 quantile for distinguishing activity levels is an arbitrary choice since there is no well-established guideline for low versus high activity level.

#### ECG data

ECG is acquired using a single lead event recorder (Preventice Solutions, Inc) and sampled at 256 Hz. ECG data were collected with the same biopatch as the accelerometer. Continuous single channel ECG signals were collected while wearing the device. Arrhythmia classification was performed using the BeatLogic platform that is cleared to identify AF episodes [5]. Our clinician has also reviewed 150 randomly selected AF events annotated by Beatlogic for further validation, out of which 139 (93%) were confirmed to be AF events. A total of 961 AF events were annotated by the algorithm. Most of the participants had less than 50 episodes of AF events throughout the 3 weeks with several participants with higher number of AF events. Figure 2 shows burden of AF for all participant and those included in the prediction algorithm analysis.

In terms of $\hbox {heart rate (HR)}$, RVR episodes are defined as having $HR>110$ $\hbox {beats per minute (BPM)}$, and non-RVR episodes with $HR \le 110$ BPM. Pre-processing, noise removal, and prediction interval extraction were performed on the synchronized recordings of ECG and accelerometer magnitude signals.

In the signal pre-processing step, a second-order Butterworth band-pass filter was applied with cutoff frequencies of 0.5 and 40 Hz to the raw ECG signal for noise removal. In the next step, a double median filter with orders equal to 0.2 and 0.6 times the sampling frequency was applied to remove baseline wandering. Peak-detection algorithm was then applied to capture the R-peaks in the ECG signals. Noise section of the signals was detected with annotations from BeatLogic platform.

Events that occurred too close to previous events were excluded to avoid overlap of prediction intervals with arrhythmia events. Events that occurred within 8 min of a noisy signal were also excluded to ensure that the prediction interval is outside the noisy signal range. We used the ECG signal to predict episodes of AF with RVR and low activity level versus all other AF episodes.

**Fig. 3:**
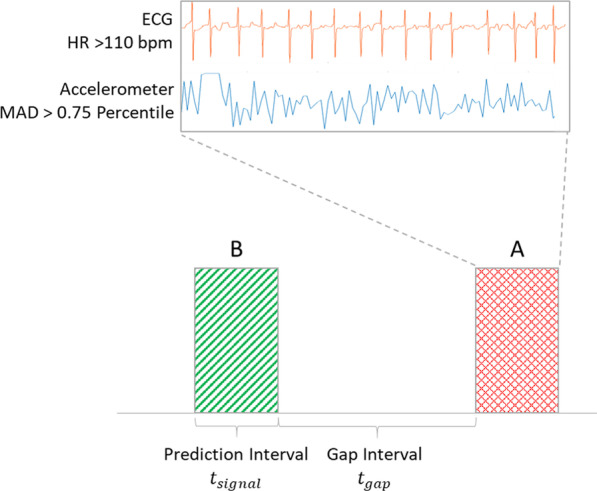
Prediction and gap intervals. Prediction and gap intervals used for prediction analysis

**Fig. 4:**
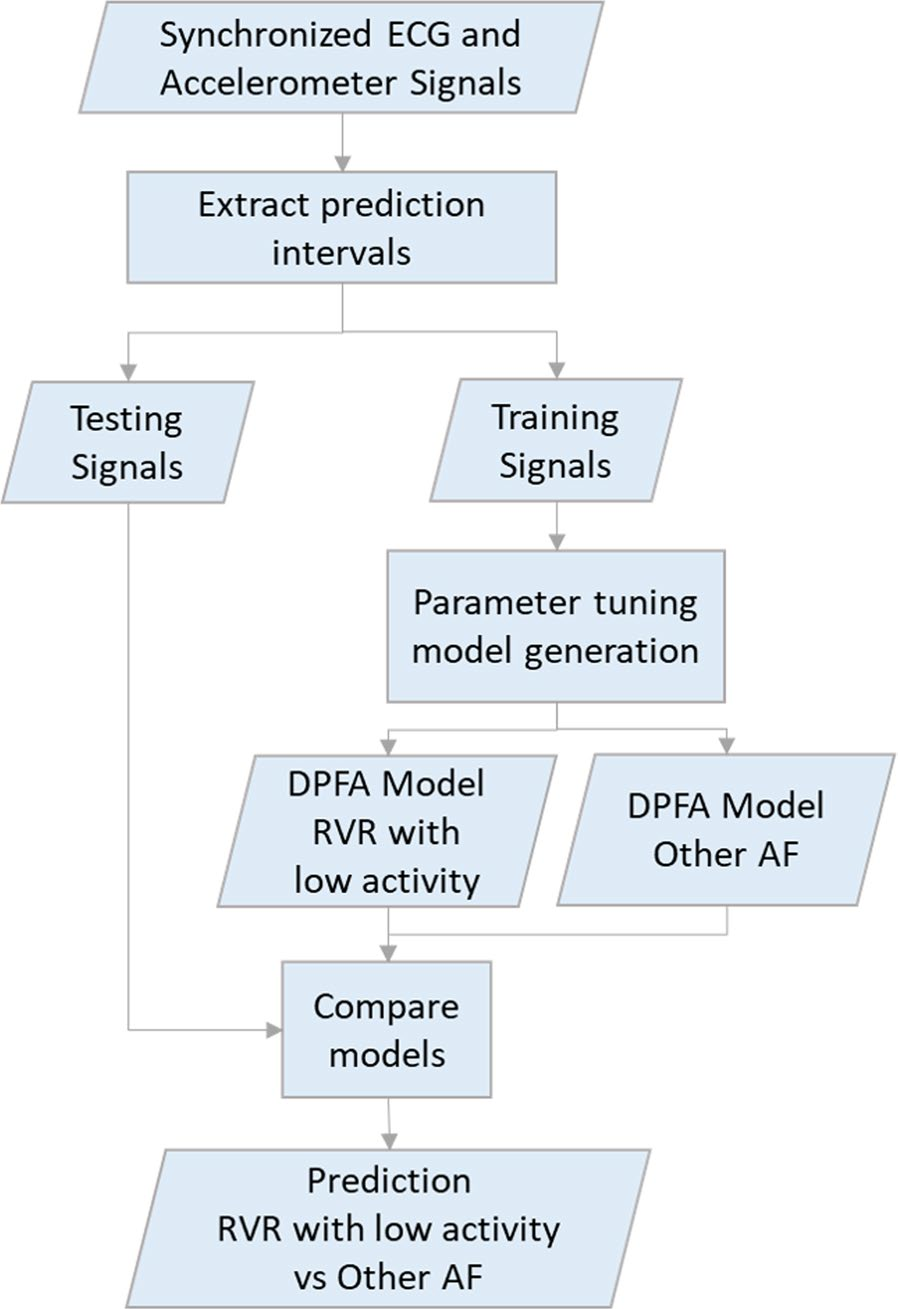
DPFA model classification. Classification of events using DPFA model

**Fig. 5:**
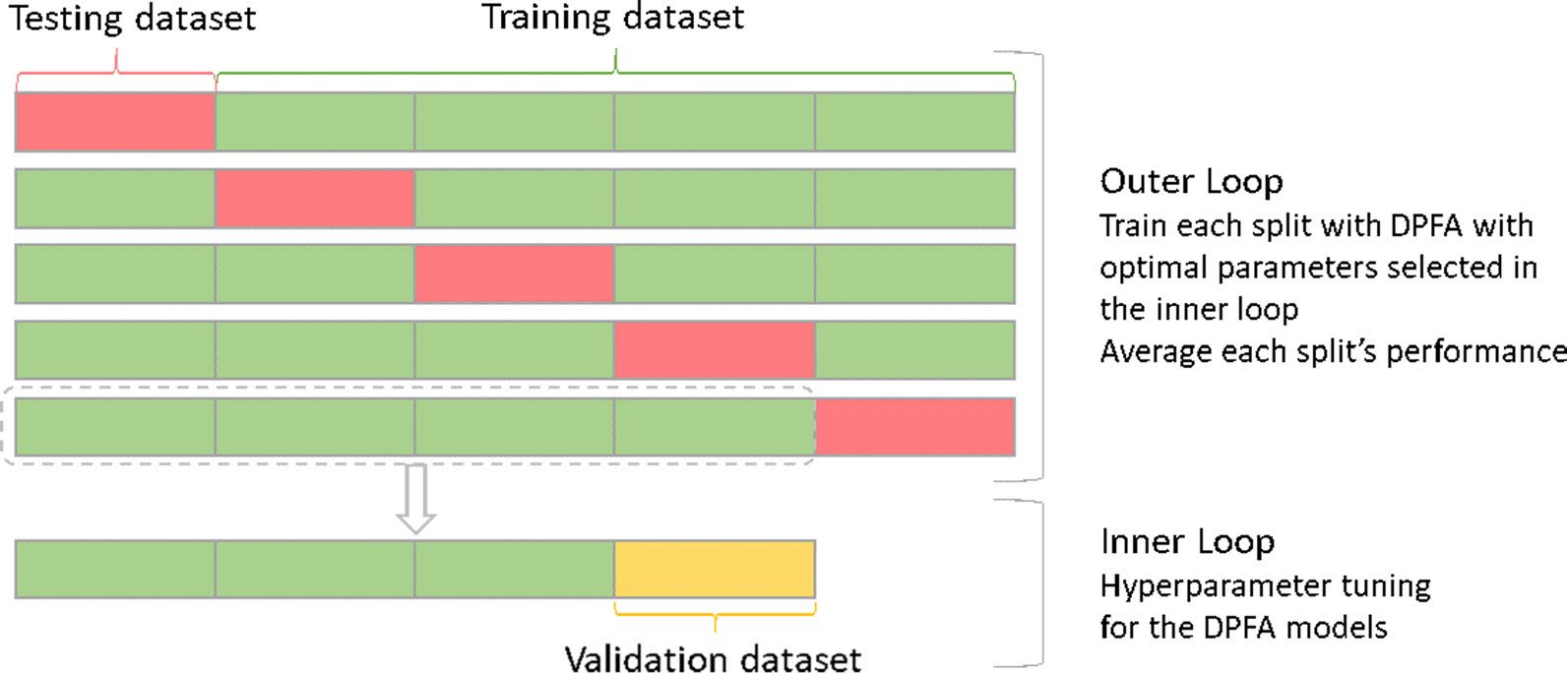
Five-fold nested cross validation

The gap interval $t_{gap}$ represents the interval in minutes before the event that is used for prediction. The signal interval $t_{signal}$ represents the length of the prediction signals. For example, prediction intervals with $t_{gap}=1$ min, $t_{signal} =2$ min are the signals that span from 3 to 1 mins before the annotated events. Figure 3 shows an example of an annotated AF episode and prediction intervals.

### DPFA model

A $\hbox {deterministic probabilistic finite-state automata (DPFA)}$ is a computational model which could be used to generate a string of letters from a fixed alphabet. Given a training sample of strings, a $\hbox {DPFA}$ could be effectively trained to summarize and reproduce the predominant rhythm patterns within the training data. In previous work, we have applied a novel $\hbox {DPFA}$-based algorithm to predict $\hbox {atrial high-rate episodes (AHREs)}$, a surrogate for AF, and $\hbox {supraventricular tachycardia (SVT)}$ events by analyzing ECG data several minutes prior to the onset of the cardiac event. In most cases the algorithm outperforms other more traditional and well-established approaches. [7].

In this study, we improved our algorithm and tested its ability to work with different types of synchronized physiological signals (i.e synchronized ECG with accelerometer magnitude signals) for arrhythmia events predictions. We used the DPFA algorithm for predicting AF episodes with RVR and low activity level versus other AF episodes using ECG and accelerometer magnitude signals prior to the event.

Each DPFA is generated in two steps (see [7] for details of the algorithm):

First in the symbolization module, one begins with the training dataset which consists of annotated ECG and accelerometer magnitude signals. From these, the algorithm extracts windows that are indicative of imminent events and others that are not. The positive (i.e., AF with RVR with low activity) and negative (i.e., all the other regions) ECG and accelerometer magnitude signals are respectively combined and transformed into probabilistic strings. Here the probabilistic strings consist of an alphabet of nine symbols $\Sigma =\{\alpha _1\beta _1,\alpha _2\beta _1,\alpha _3\beta _1,\alpha _1\beta _2,\alpha _2\beta _2,\alpha _3\beta _2,\alpha _1\beta _3,\alpha _2\beta _3,\alpha _3\beta _3\}$ where $\alpha _i$ correspond to different activity levels and $\beta _j$ correspond to different ECG morphology types within each window, and the probabilities

3 $$\begin{aligned} {\mathbf {p}}(\alpha _i\beta _j)={\mathbf {p}}(\alpha _i){\mathbf {p}}(\beta _j) \end{aligned}$$

are computed by assigning probabilities to $\alpha _i$ and $\beta _j$ independently. The individual probabilities ${\mathbf {p}}(\alpha _i)$ and ${\mathbf {p}}(\beta _j)$ are obtained from the signal values $x_t$ over discrete time windows *t* via soft-thresholding

4 $$\begin{aligned} \left\{ \begin{array}{ccl}{\mathbf {p}}_1&{}=&{}\psi _1(x_t)\\ {\mathbf {p}}_2&{}=&{}\big (1-\psi _1(x_t)\big )\cdot \psi _2(x_t)\\ {\mathbf {p}}_3&{}=&{}1-{\mathbf {p}}_1-{\mathbf {p}}_2,\end{array}\right. \end{aligned}$$

where the soft-thresholding functions are chosen to be piecewise linear functions $\psi _1,\psi _2:[0,1]\rightarrow [0,1]$ of the form

5 $$\begin{aligned} \psi _j(x)={\left\{ \begin{array}{ll} 1 &{} \text { if } x> b_j \\ \frac{x-a_j}{b_j-a_j} &{} \text { if } a_j\le x\le b_j \\ 0 &{} \text { if } x< a_j. \end{array}\right. } \end{aligned}$$

The parameters $a_1, b_1, a_2, b_2$ were all tuned in the training step and set at

6 $$\begin{aligned} \left\{ \begin{array}{lcc} a_1&{}=&{}0.03\\ b_1&{}=&{}0.05\\ a_2&{}=&{}0.01\\ b_2&{}=&{}0.02 \end{array}\right. \quad \text {for activity}\alpha _i~\text {and}\quad \left\{ \begin{array}{lcc} a_1&{}=&{}0.5\\ b_1&{}=&{}0.7\\ a_2&{}=&{}0.025\\ b_2&{}=&{}0.05 \end{array}\right. \quad \text {for ECG }\beta _j. \end{aligned}$$

based on a grid search while running the inner loops of the nested cross validation.

Then the DPFA generation module constructs the positive (AF with RVR and low activity) DPFA $M_+$ and the negative (all other AF episodes) DPFA $M_-$ respectively from the positive and negative probabilistic strings, by first constructing the frequency prefix trees $T_+$ and $T_-$, followed by the largest suffix merging algorithm $T_+\rightarrow M_+$ and $T_-\rightarrow M_-$. The frequency prefix trees are tree-like automata $T=\langle Q_0,\Sigma ,\varepsilon ,\mathrm {Freq}\rangle$ with initial state $\varepsilon$ whose state space $Q_0$ consists of strings in the alphabet $\Sigma$ with non-zero frequency, and transition function given by concatenation of strings. The largest suffix merging algorithm selects those states $q\in Q_0$ of the frequency prefix tree *T* with sufficiently high frequency to be in the state space *M*, and then defines the transition state $T(q,\alpha _i\beta _j)$ to be the largest suffix of $q\alpha _i\beta _j$ that is itself contained in the state space of *M*. See [7] for further details.

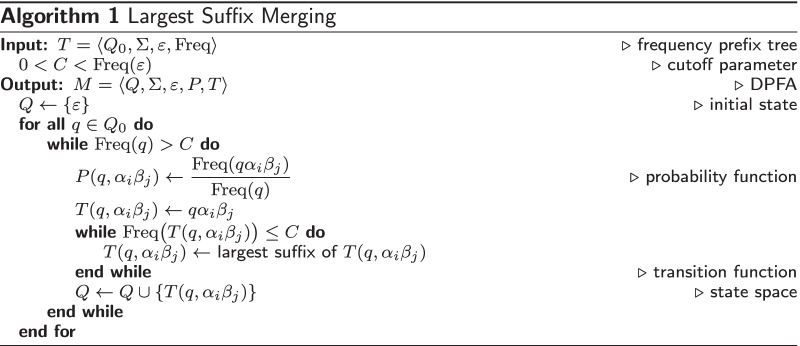



The classification scheme contains a training phase and a testing phase. In the training phase, the algorithm learns the DPFA $M_+$ and $M_-$ for the positive and negative classes respectively. Then in the testing phase, we classify a given synchronized ECG-accelerometer magnitude signal from the test dataset by comparing the goodness-of-fit with the DPFA $M_+$ and $M_-$ to predict episodes of AF with RVR with low activity Fig. 4.

### Data partition

A total of 961 episodes of AF events were annotated for the analysis. We used 5 fold nested cross validation for parameter tuning. Figure 5 shows how data was partitioned in the analysis. The 961 episodes were split into 5 folds, 4 folds were used for training and the remaining fold was used for testing. Within the inner loop, the training data had a total of 4 folds, 3 folds were used for parameter tuning and the remaining fold for validations. In the outer loop, the DPFA models were generated using all 4 folds of the training data with hyper-parameters tuned during the inner loop. Predictions were performed on the testing data in each split and then averaged.

## Results

During the study period, we have recruited 45 patients with AF, of whom 31 had both ECG and accelerometer data. We excluded patients with all AF episodes of duration less than 30 seconds and those with less than 5 episodes of AF. There was a total of 292 episodes of AF with RVR compared to 669 episodes of AF with controlled heart rate. There were 116 episodes in $>75$ percentile activity level in the RVR group and 124 episodes in the non-RVR group based on MAD relative to total group. The distribution of high activity based on heart rates above 110 BPM are summarized in Table 2. The 45 participants in the study had a median of 22 days of total wear time (range 2–31 days). The median overall AF burden was $38.4\%$ ($\hbox {interquartile range (IQR)}$ 4.9–86.0%).

Among the 961 annotated AF events, 292 of them met the criterion for RVR episode, among which 176 episodes had low activity level and the remaining 116 episodes had high activity level based on MAD relative to the entire group activity level. For the rest of the annotated AF events, 669 of them were non-RVR episodes, among which 545 episodes had low activity and the remaining 124 episodes had high activity level. Table 2 summarizes the number of annotated AF episodes by activity and $\hbox {HR}$ levels.

A total of 18 patients are included in the final DPFA model. They had a median of 22 days of total wear time (range 6–31 days). The median overall AF burden for the 18 patients was $19.7\%$ ($\hbox {IQR}$ 10.5–62.3%). Two participants had an AF burden $> 90\%$ over the duration of the study. The mean heart rate was $99.0\pm 19.4$ BPM. The burden of AF for study participants is summarized in Fig. 2.

Different combinations of signal intervals (i.e., $t_{signal}=0.5,1.0,2.0$ min and gap intervals (i.e., $t_{gap}=0.5,1.0,2.0,2.5,3.5,4.0,4.5$ min) up to 5 min before the AF events were used for prediction. For a given model based on various gap intervals, the threshold can be adjusted to optimize different parameters, i.e., a more sensitive model versus a more specific model. In Table 3, we report the threshold, sensitivity, specificity, precision, and other relevant data based on each gap interval.

**Fig. 6:**
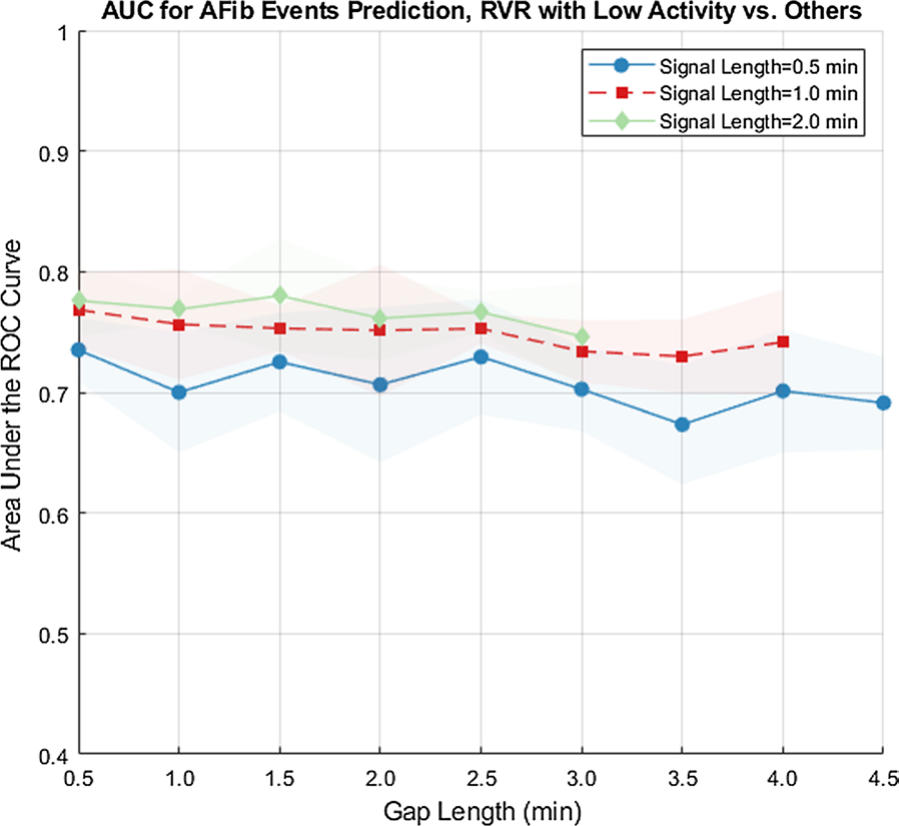
AUC for AFib events predictions, RVR with low activity versus other by group level MAD. AUC under the ROC curve for various prediction intervals, for AF duration of at least 30 seconds nested cross validation, activity grouped by group level MAD

**Fig. 7:**
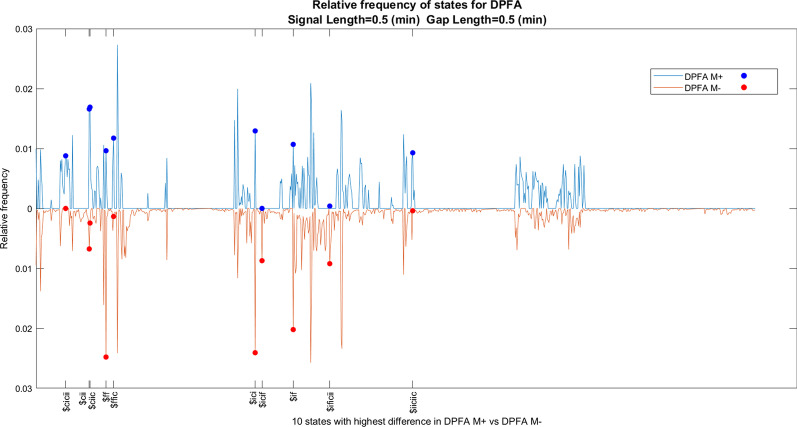
Relative frequency of states for DPFA models, signal length $=0.5$ (mins), gap Length $= 0.5$ (mins)

### Prediction results

We limited the events to those that lasted for at least 30 seconds to investigate the effects of AF duration on predicting episodes of AF with RVR with low activity. As the time to the event increases, the mean prediction performance gradually declines while the variance increases. Using the MAD threshold relative to the whole group, the average AUC for 0.5 min-long prediction intervals is $0.71 \pm 0.04$, 1 min-long is $0.75 \pm 0.03$ and 2 minute-long is $0.77 \pm 0.03$ (Fig. 6). The 2 minute-long prediction intervals also showed the highest AUC around 0.75 with smallest standard deviations. Prediction results for various gap intervals and prediction intervals are shown in Table 3.

### DPFA model interpretation

In addition to the superior performance, another major advantage of our approach is the interpretability of the underlying DPFA models. Indeed the DPFA models could be understood by first calculating the relative frequencies of the states, and then identifying the most prevalent rhythm patterns represented by the states within the DPFA model constructed from each class. This could be achieved via standard techniques by first extracting the normalized leading eigenvector of the transition matrix of the DPFA model, and then taking the average of the relative frequencies across the five cross-validation folds. For example, by comparing the two DPFA models $$M_+$$ and $$M_-$$ with signal length $$=0.5$$ (mins) and gap length $$=0.5$$ (mins), this approach yields the following five rhythm patterns as showing the most significant difference between $$M_+$$ and $$M_-$$: $ff (which corresponds to the rhythm pattern $$[\alpha _3\beta _2,\alpha _3\beta _2]$$, where $$\alpha _3\beta _2$$ corresponds to a single window with activity level $$\alpha _3$$ and ECG type $$\beta _2$$ as defined by equation (3, 4, 5, 6)), $ciic, $ici, $ffic, and $cii. Here for ease of presentation we have switched to an alphabet consisting of single-letter symbols, the two alphabets correspond to each other as follows

**Table:** 

Old alphabet	$\alpha _1\beta _1$	$\alpha _2\beta _1$	$\alpha _3\beta _1$	$\alpha _1\beta _2$	$\alpha _2\beta _2$	$\alpha _3\beta _2$	$\alpha _1\beta _3$	$\alpha _2\beta _3$	$\alpha _3\beta _3$
New alphabet	a	b	c	d	e	f	g	h	i

**Table.3:** Prediction results for various gap intervals and prediction intervals

Gap interval (min)	Prediction interval (min)	AUC mean (Std)	Sensitivity mean (Std)	Specificity mean (Std)	Accuracy mean (Std)
0.5	0.5	0.735(0.026)	0.552(0.049)	0.808(0.104)	0.806(0.027)
1	0.5	0.700(0.050)	0.546(0.075)	0.797(0.085)	0.811(0.035)
1.5	0.5	0.725(0.041)	0.545(0.098)	0.804(0.078)	0.810(0.024)
2	0.5	0.706(0.064)	0.527(0.111)	0.810(0.084)	0.799(0.027)
2.5	0.5	0.729(0.048)	0.521(0.103)	0.845(0.065)	0.781(0.062)
3	0.5	0.703(0.035)	0.511(0.115)	0.790(0.091)	0.758(0.088)
3.5	0.5	0.673(0.049)	0.527(0.121)	0.742(0.092)	0.793(0.059)
4	0.5	0.701(0.051)	0.521(0.115)	0.802(0.110)	0.718(0.068)
4.5	0.5	0.691(0.038)	0.520(0.145)	0.790(0.073)	0.785(0.060)
0.5	1	0.768(0.031)	0.541(0.041)	0.850(0.081)	0.779(0.109)
1	1	0.756(0.046)	0.524(0.074)	0.838(0.097)	0.841(0.019)
1.5	1	0.753(0.019)	0.545(0.092)	0.838(0.104)	0.826(0.021)
2	1	0.751(0.054)	0.488(0.094)	0.864(0.073)	0.828(0.043)
2.5	1	0.753(0.012)	0.585(0.102)	0.805(0.135)	0.827(0.040)
3	1	0.734(0.026)	0.569(0.116)	0.767(0.128)	0.783(0.106)
3.5	1	0.730(0.030)	0.573(0.108)	0.809(0.117)	0.784(0.103)
4	1	0.742(0.043)	0.567(0.101)	0.819(0.132)	0.775(0.117)
0.5	2	0.776(0.029)	0.547(0.087)	0.838(0.074)	0.836(0.047)
1	2	0.769(0.011)	0.584(0.141)	0.824(0.117)	0.818(0.044)
1.5	2	0.780(0.047)	0.572(0.119)	0.869(0.088)	0.814(0.071)
2	2	0.761(0.034)	0.603(0.090)	0.782(0.150)	0.826(0.038)
2.5	2	0.766(0.017)	0.572(0.081)	0.820(0.098)	0.790(0.076)
3	2	0.746(0.044)	0.586(0.114)	0.777(0.129)	0.779(0.124)

**Table.4:** Five states with most difference between the relative frequencies in DPFA $M_+$ versus DPFA $M_-$ for various gap intervals and prediction intervals

Gap interval (min)	Prediction interval (min)	State1	State2	State3	State4	State5
0.5	0.5	$ff	$ciic	$ici	$ffic	$cii
1	0.5	$i	$ciic	$cii	$icii	$iffff
1.5	0.5	$iiiciici	$iiiciii	$ciic	$icci	$ciici
2	0.5	$ff	$cii	$ici	$cifi	$f
2.5	0.5	$ficific	$ific	$ificic	$iiiciii	$iffi
3	0.5	$fi	$cii	$ficific	$ciic	$icciic
3.5	0.5	$ficific	$iccifici	$iiiciicic	$iccii	$icific
4	0.5	$ff	$cii	$i	$fi	$icc
4.5	0.5	$cifici	$icif	$cific	$cifi	$fici
0.5	1	$iicciiiiciii	$iicciiiic	$iicciiiicii	$iicciiiici	$iicciiicii
1	1	$ificificifici	$ificificific	$iicifii	$iicifiic	$ciicicii
1.5	1	$ficifici	$cific	$ciici	$cif	$ficificificif
2	1	$ciic	$cii	$cific	$ciii	$iccii
2.5	1	$cific	$ciii	$icif	$ciic	$cicici
3	1	$ificiiic	$ii	$cificif	$ficif	$iccic
3.5	1	$i	$ic	$f	$if	$iiciici
4	1	$ificificifici	$icci	$iiccificii	$iiccifii	$ificificific
0.5	2	$ificificificifici	$iiiiciiiicii	$iiiiciiciicii	$iiiiciiiiciii	$ificific
1	2	$iiiiciicii	$ificific	$ifiic	$ificificificif	$ificificifici
1.5	2	$ificificifi	$ificificificific	$ii	$ficifici	$ificificificifici
2	2	$ici	$icifi	$icif	$ffff	$iccificifi
2.5	2	$iiccif	$ificificific	$iicc	$iiccic	$iiciiii
3	2	$iccii	$fici	$ficific	$ficif	$icif

and we use the $ symbol to denote the empty string. Please see Fig. 7 for a plot of the relative frequencies of all the states and the ten most significant rhythm patterns with signal length $$=0.5$$ (mins) and gap length $$=0.5$$ (mins). Please also see Table 4 for the five states with most difference between the relative frequencies in DPFA $$M_+$$ versus DPFA $$M_-$$ for various gap intervals and prediction intervals.

## Discussion

This study in patients with AF showed that our novel DPFA algorithm can predict the onset of AF with RVR associated with low levels of activity with AUC around 0.75 for intervals up to 4.5 min. before the onset of the event. This is the first study to evaluate the performance of prediction algorithms using the ECG along with accelerometer data. Prediction of AF with rapid rates can result in personalized treatment options for prevention and management of AF episodes that are likely to be clinically significant. Our algorithm can help to distinguish between AF with RVR episodes that occur unexpectedly at low levels of activity, thus more likely to be clinically significant.

The machine learning techniques have shown impressive capability to analyze massive amounts of data and are an effective method for classification of arrhythmias using ECG data. Despite being most commonly used in many areas, deep learning algorithms have the drawback that their architecture represents a “Black Box” [8]. Their lack of transparency limits their clinical adoption, makes mechanistic interpretation difficult and reduces their trustworthiness. Further, the disclosure of meaningful details about medical treatment to patients requires the doctors to grasp the fundamental inner workings of the devices they use to some degree [9]. Explainability may also be required to justify the clinical validation of machine learning algorithms in prospective studies and randomized clinical trials [10]. Our DPFA model represents a novel and explainable algorithm. This allows others to validate our algorithm in a diverse population of patients and adopt it clinically if proven to be effective. Previous studies have used the features extracted from the accelerometer data to give an overall impression of patient activity over a period of time. However, these features can not fully capture the variability in daily activity. It also misses the features within the activity signal that can provide important physiological insights about the patient’s condition [11]. Our approach shows that using the one-dimensional accelerometer magnitude from the tri-axial raw accelerometer data can provide important information that can augment the interpretation of the ECG and accelerometer signals beyond the traditional features extracted from the signals.

Most methods for physiological data analysis depend heavily on pre-processing. However, these methods tend to be less effective on noisy data, such as data collected in real-time or in outpatient settings. Therefore, it is desirable to introduce new methods that require minimal pre-processing to analyze such data, thus allowing these insights to be applied to automated clinical decision making. In the previous study, the DPFA method has showed to be particularly useful for the real-world noisy data. Our algorithm does not rely on peak detection, requires minimal pre-processing and leads to good performance in the setting of noisy ECG signal. This provides a significant advantage over existing algorithms when it is necessary to perform rapid analysis in highly noisy environments. Thus DPFA algorithm can be useful for signals captured by portable devices which are prone to noise.

Prediction can only be helpful if it results in specific action that can impact clinical outcomes. In recent years, an increasing number of portable devices have been developed to monitor the physiological signals. Our algorithm provides a possibility for real-time prediction of clinically meaningful arrhythmias using ECG signal together with accelerometer signals with enough lag time for medical interventions.

This is the first algorithm that can identify features within the ECG that are capable of detecting and predicting periods of AF with RVR that are not associated with high activity levels. This allows clinicians to distinguish between clinically significant periods of AF and other periods. This has significant implications for the appropriate treatment of patients with AF. Identifying physiologically significant episodes of AF allows an opportunity to deliver just-in-time treatments that can be tailored for each individual while avoiding the side effects related to daily medications.

### Limitations

The first limitation of our study is the sample size. Our algorithm was validated in a small subset of patients with AF. Although the number of patients included in the study was small, there were numerous episodes available for training our algorithm. The validity and generalizability of our algorithm needs to be tested in a more diverse group of patients with AF. We sought to mediate the impact of patient subtypes by leveraging a large number of episodes of AF in our patient population; however, future work incorporating medication status, co-morbidities and other factors would allow for measuring algorithm performance within specific patient subsets while ensuring equal representation in the training dataset.

The aim of our project was to predict AF with rapid ventricular rates and low activity levels. Worsening AF symptom severity is associated with reduced daily activity [12]. The second limitation of the study is a lack of patient-reported symptoms during periods of AF with RVR and low activity. Therefore, it is difficult to ascertain symptom severity during these episodes. Future studies with frequent momentary assessment of symptoms are needed to determine the relationship between symptoms and AF with RVR episodes and low activity.

The third limitation is the interpretability of the activity threshold. In the study, an arbitrary choice of 0.75 quantile MAD based on activity of the entire group is used for classifying activity level. However, participants might have large differences in activity level. To account for these differences, MAD threshold relative to individual participants is also used for analysis. The average $\hbox {AUC}$ for 0.5 min-long prediction intervals is $0.74 \pm 0.02$, 1 min-long is $0.75 \pm 0.01$ and 2 min-long is $0.78 \pm 0.01$. The 2 min-long prediction intervals showed most consistent results with $\hbox {AUC}$ around 0.75 and smallest standard deviations. When $t_{gap}$ is less than 1.5 min, the $\hbox {AUC}$ ranges between 0.74-0.80. The $\hbox {AUC}$ is above 0.69 for all prediction intervals. We did not distinguish between different types of activity. It is possible that certain forms of exercise (e.g., stationary cycling) may not be accounted for in the present analysis. The MAD threshold used in the study is a relative MAD threshold instead of an absolute MAD threshold. Studies have investigated different types of activities with their MAD thresholds. However these studies [6] used accelerometer sensors attached to the hip or wrist regions while our sensors are attached to the chest which make our MAD measurements incomparable with these thresholds. There are several studies that investigate activity classification using chest mounted tri-axial accelerometer devices [13–16] with signal processing and machine learning techniques, however they did not state thresholds of activities with MAD metric. We tried to minimize this limitation by using both intra-individual and sample-level activity distributions for the threshold. However, in the future if accelerometer data can be collected with similar sensors and attached to wrists, the prediction results can be better generalized. Both the accelerometer data and ECG data are collected as continuous signals with timestamp when the device is attached to the participant, so the non-wear time information is available and automatically removed, since our study uses synchronized accelerometer and ECG signals. The non-wear time is unlikely to affect our results on prediction of RVR with low activity level. But for future studies it could be helpful to know the reason why the patient removed the device and if it was a consequence of potential events. To this end, we plan to standardize and automate the annotation process by intelligently mining the training data. We are hopeful that such efforts will continue to improve of the performance of the algorithm and further expand the scope of our approach.

## Conclusion

In this paper, a DPFA based method is performed for predicting the onset of AF with RVR associated with low levels of activity up to 4.5 min prior to the onset of the events. The proposed algorithm takes input from two different types of synchronized physiological signals, constructs the state space and the underlying transition probabilities directly from the data input. The model achieved an overall AUC around 0.7–0.8 depending on the lengths and gap sizes of the prediction intervals. Although the database is relatively small in terms of number of participants, each participant has up to 3 weeks of continuous synchronized ECG and accelerometer data. The performance of the proposed DPFA algorithm does not depend on pre-processing the data. With a larger dataset and additional validation, our algorithm could enable the development of just-in-time intervention alert system on wearable portable devices that could reduce the morbidity and mortality associated with AF and other similar arrhythmias.

## Data Availability

The datasets used and/or analysed during the current study are available from the corresponding author on reasonable request.

## Abbreviations

AF: Atrial fibrillation
AHRE: Atrial high-rate episode
AUC: Area under the ROC curve
BPM: Beats per minute
DPFA: Deterministic probabilistic finite-state automata
ECG: Electrocardiogram
HR: Heart rate
IQR: Interquartile range
MAD: Mean amplitude deviation
RVR: Rapid and irregular ventricular rate
SVT: Supraventricular tachycardia
